# Evaluation of a Digital Decision Support System to Integrate Type 2 Diabetes Mellitus and Periodontitis Care: Case-Vignette Study in Simulated Environments

**DOI:** 10.2196/46381

**Published:** 2023-10-02

**Authors:** Olivier Kalmus, Kirsten Smits, Max Seitz, Christian Haux, Bernt-Peter Robra, Stefan Listl

**Affiliations:** 1 Section for Translational Health Economics Heidelberg University Hospital, Department of Conservative Dentistry Heidelberg University Heidelberg Germany; 2 Department of Dentistry, Quality and Safety of Oral Healthcare Radboud Institute for Health Sciences Radboud University Medical Center Nijmegen Netherlands; 3 Institute of Medical Informatics Heidelberg University Heidelberg Germany; 4 Institute of Social Medicine and Health Systems Research Otto-von-Guericke-University Magdeburg Germany

**Keywords:** digital health, integrated care, decision support, oral health, diabetes, periodontitis, decision support, oral care, type 2 diabetes, evaluation, survey, hemoglobin, diagnostic device, telemedicine

## Abstract

**Background:**

As highlighted by the recent World Health Organization Oral Health Resolution, there is an urgent need to better integrate primary and oral health care. Despite evidence and guidelines substantiating the relevance of integrating type 2 diabetes mellitus (T2DM) and periodontitis care, the fragmentation of primary and oral health care persists.

**Objective:**

This paper reports on the evaluation of a prototype digital decision support system (DSS) that was developed to enhance the integration of T2DM and periodontitis care.

**Methods:**

The effects of the prototype DSS were assessed in web-based simulated environments, using 2 different sets of case vignettes in combination with evaluation surveys among 202 general dental practitioners (GDPs) and 206 general practitioners (GPs). Each participant evaluated 3 vignettes, one of which, chosen at random, was assisted by the DSS. Logistic regression analyses were conducted at the participant and case levels.

**Results:**

Under DSS assistance, GPs had 8.3 (95% CI 4.32-16.03) times higher odds of recommending a GDP visit. There was no significant impact of DSS assistance on GP advice about common risk factors for T2DM and periodontal disease. GDPs had 4.3 (95% CI 2.08-9.04) times higher odds of recommending a GP visit, 1.6 (95% CI 1.03-2.33) times higher odds of giving advice on disease correlations, and 3.2 (95% CI 1.63-6.35) times higher odds of asking patients about their glycated hemoglobin value.

**Conclusions:**

The findings of this study provide a proof of concept for a digital DSS to integrate T2DM and periodontal care. Future updating and testing is warranted to continuously enhance the functionalities of the DSS in terms of interoperability with various types of data sources and diagnostic devices; incorporation of other (oral) health dimensions; application in various settings, including via telemedicine; and further customization of end-user interfaces.

## Introduction

As highlighted by the recent World Health Organization Oral Health Resolution, there is an urgent need to better integrate primary and oral health care [1,2]. In particular, there is mounting evidence of a bidirectional relationship between type 2 diabetes mellitus (T2DM) and periodontitis [3,4]. Individuals with T2DM are more likely to develop severe periodontitis [5]. Conversely, individuals with periodontitis are at increased risk of developing T2DM [6]. Considering that the number of people with undiagnosed T2DM is expected to be rather high, identifying these patients should be a priority in health care systems [7]. Also, individuals with both T2DM and periodontitis face more difficulties achieving good glycemic control and develop other T2DM-related complications more frequently [8,9]. Consequently, the International Diabetes Federation and the European Federation of Periodontology have developed guidelines for the integration of T2DM and periodontal care [10]. However, treatment of T2DM and periodontitis is still often provided by separate providers with little integration on the health systems and daily practice levels [1,2]. Improving cooperation and information exchange between primary and oral health care could offer health and economic benefits. For example, previous studies have demonstrated the potential of screening instruments for prediabetes or diabetes and periodontitis risk as a basis for more integrated care concepts [11-13].

Digital health solutions provide unique opportunities to help integrate oral health and primary health care. To this end, the German Innovation Fund project Dent@Prevent has developed a prototype digital decision support system (DSS) that aims to enhance the interdisciplinary integration of T2DM and periodontal care among general practitioners (GPs) and general dental practitioners (GDPs) [14]. DSSs have been deployed primarily in clinical care but are becoming more frequent in other areas as well [15]. They have been shown to be useful in improving practitioner performance and care processes [16]. However, the exact criteria for a successful implementation are not fully understood and are dependent on various factors, including context, users’ characteristics, and integration in the workflow [15]. The potential effectiveness of a DSS must therefore be comprehensively evaluated before it can be rolled out in clinical contexts and cyclically updated on a larger scale.

Case vignettes have been shown to be a useful tool to assess health care providers’ decision-making and their approach to diagnosis and treatment of patients [17]. While standardized patients are considered the gold standard when trying to assess clinical care, case vignettes offer the advantage of being more affordable, faster to implement, and deployable in a wide range of settings [18,19]. Additionally, they have been shown to be a valid tool for measuring the process of care compared with actual clinical practice, chart abstraction, and standardized patients [18]. They also offer the opportunity to control for case-mix variation [20].

The purpose of this study was to evaluate, through case vignettes, a prototype DSS that was designed to improve cooperation between GPs and GDPs. We hypothesized that DSS assistance would facilitate more interdisciplinary cooperation and thus offer a path to better care. This could represent a contribution to the movement to improve the integration of dental and general care.

## Methods

### DSS Functionality

The prototype DSS was designed to operate on patient-reported data alone: through a previously validated mobile app [21], patients fill in a survey based on the Periodontitis Risk Score [22], the Finnish Diabetes Risk Score (FINDRISC) questionnaire [23], and a basic anamnesis. The DSS then calculates the patients’ risk for periodontitis and T2DM. For patients with a calculated high risk, the DSS prompts the practitioners to mention the bidirectional relationship between T2DM and periodontitis and their common risk factors and suggests recommending a visit to the GP or GDP to check for potential T2DM or periodontitis, respectively. GDPs are also encouraged to ask for glycated hemoglobin (HbA_1c_) values from patients with known diabetes and take these values into account when planning periodontal treatment. A more technical description of the software development is out of the scope of this (proof-of-concept) study and will be published separately.

### Data Collection and Sampling

Drawing from the advice of 3 experts (each with a different yet complementary scientific background in medical, dental, or social medicine), we developed 2 sets of case vignettes: one set for the GP setting and another set for the GDP setting. Vignette attributes were chosen based on clinical practice, and the attribute values were chosen based on the functionalities and actions of the DSS. All other information necessary for treatment was kept fixed for all cases as part of the vignette. Textbox 1 shows all attributes and fixed characteristics retained in the vignette creation.

**Vignette attributes and characteristics.**

**GP vignettes**

- Age (kept fixed for all cases)
- Sex
- Reason for visit
- Complaints and symptoms
- Duration of symptoms
- Past examinations and lab results
- Medication
- Smoking behavior
- Diet
- Physical activity
- Family anamnesis
- Physical examination results
- Technical examination results
- Blood laboratory results

**GDP vignettes**

- Age (kept fixed for all cases)
- Sex
- Reason for visit
- Complaints and symptoms
- General condition
- Picture of the mouth
- X-ray
- Chronic conditions
- Infectious diseases (kept fixed for all cases)
- Medication
- Allergies (kept fixed for all cases)
- Pregnancy (kept fixed for all cases)
- Smoking behavior
- Clinical examination results
- Periodontal screening and assessment
- Vitality test results (kept fixed for all cases)
- Oral hygiene behavior (kept fixed for all cases)
- Diet
- General health problems
- Physical activity
- Family anamnesis
- HbA_1c_ value

We then developed an interactive survey relying on closed-ended questions to evaluate the treatment of the selected vignettes. The vignette presentation and the survey were designed in a way to reflect everyday practice as closely as possible and avoid any framing of study participants. Specific information was only presented if the participant chose the corresponding option. The vignettes were piloted in 2 focus group discussions, 1 with GPs and 1 with GDPs, and adjustments were made when necessary. This yielded 3 vignettes for GPs and 6 vignettes for GDPs, representing the spectrum of cases relevant to the DSS. Textbox 2 presents the final GDP cases and Textbox 3 the final GDP cases.

**GP vignette case descriptions.**

**Case A: Undiagnosed diabetes patient with high periodontitis risk**

Patient A is coming for a physical check-up. She mentions weight loss, fatigue, and thirst. She exhibits all common risk factors for type 2 diabetes mellitus (T2DM) and periodontitis. There is a history of diabetes and heart disease in her family. Her lab results confirm a T2DM diagnosis.

**Case B: Diabetes patient in long term treatment with high periodontitis risk**

Patient B is coming for a scheduled visit part of the diabetes disease-management program. She does not have any concerns. She exhibits all common risk factors for periodontitis. Her lab results show that treatment is not improving her glycated hemoglobin (HbA_1c_) value.

**Case C: Diabetes patient starting treatment with medium periodontitis risk**

Patient C was newly diagnosed with T2DM and is coming for the first treatment visit. She does not have any concerns. She seems healthy overall and does not exhibit the common risk factors for periodontitis. Her lab results show a very high HbA_1c_ value.

**GDP vignette case descriptions.**

**Case A (female)**

- Periodontitis
- High diabetes risk
- Coming for a regular check-up
- Undiagnosed and untreated periodontitis
- Exhibits all common risk factors for type 2 diabetes mellitus (T2DM)

**Case B (male)**

- No periodontitis
- High diabetes risk
- Coming for a regular check-up
- Does not have any concerns
- Oral health and hygiene are good
- Exhibits all common risk factors for T2DM

**Case C (female)**

- Undergoing periodontal treatment
- Known T2DM
- Coming for a scheduled periodontal treatment
- Treatment so far has not led to any improvement in periodontal health
- Previously diagnosed with T2DM but does not exhibit the common risk factors
- Does not know her HbA_1c_ value

**Case D (male)**

- Periodontitis
- Known T2DM
- Coming for a regular check-up
- Undiagnosed and untreated periodontitis
- Previously diagnosed with T2DM and exhibits the common risk factors; taking oral antidiabetics
- HbA_1c_ value within the desired range

**Case E (female)**

- No periodontitis
- Known T2DM
- Coming for a regular check-up
- Does not have any concerns
- Oral health and hygiene are good
- Previously diagnosed with T2DM; does not exhibit the common risk factors
- HbA_1c_ value exceeds the desired limit

**Case F (male)**

- Undergoing periodontal treatment
- Known T2DM
- Coming for a scheduled periodontal treatment
- Treatment so far has not led to any improvement in periodontal health
- Previously diagnosed with T2DM and exhibits the common risk factors; taking oral antidiabetics
- HbA_1c_ value is very high

**Figure 1:**
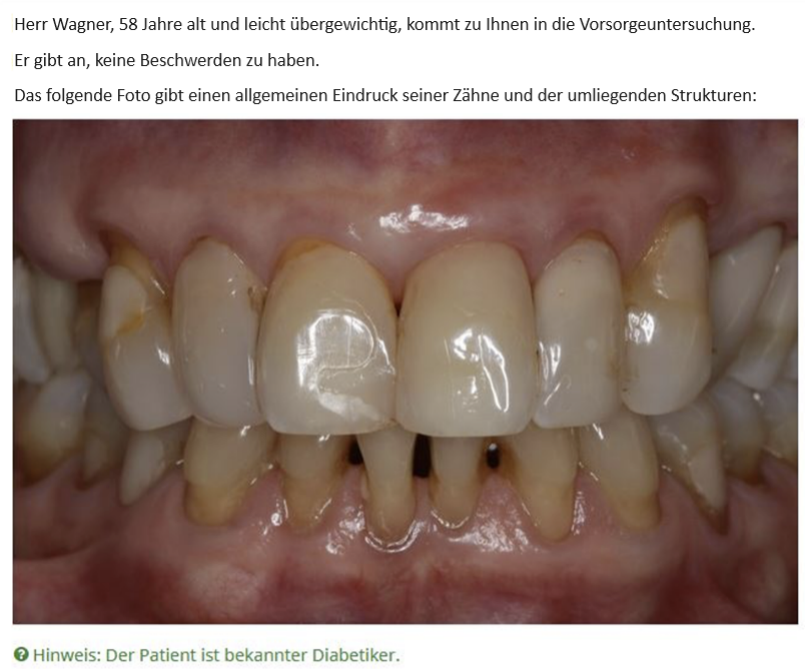
General dental practitioner vignette example with decision support system hint in green. The patient is male, aged 58 years, and slightly overweight; he is coming for a dental checkup. He does not report any concerns. The picture gives an overview of his dental situation. The text in green is only shown when decision support system assistance is active; all the text in black is shown with and without decision support system assistance.

**Figure 2:**
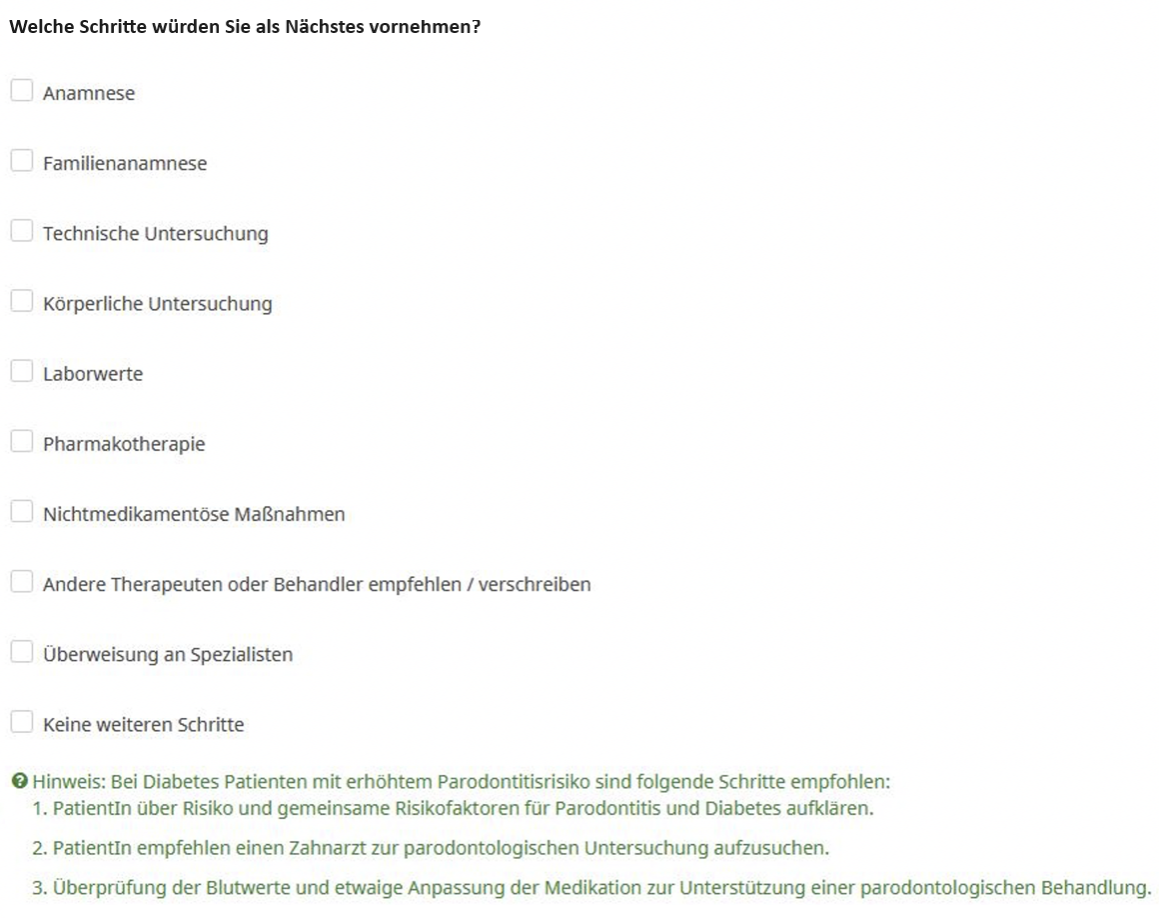
General practitioner vignette main evaluation question with decision support system hints in green. Participants were asked to choose their next steps from a multiple choice list including anamnesis, examination, therapy, referral, and “no further steps.” The text in green is only shown when decision support system assistance is active; all the text in black is shown with and without decision support system assistance.

The study was implemented using the web-based LimeSurvey platform (LimeSurvey GmbH). Due to the lack of comparable studies, we based the sample size calculation on a “medium effect,” which was estimated at around 200 GPs and 200 GDPs. Participants were recruited through the market research company DocCheck Community GmbH. No specific targeting criteria were applied; the only exclusion criterion was not actively practicing medical or dental care in Germany according to information from DocCheck (study participants were included or excluded through verification of occupation). Each participant evaluated 3 vignettes; in the case of GDPs, these were 3 randomly selected vignettes. All participants received DSS assistance for 1 of the 3 vignettes, chosen and presented at random. Figures 1 and 2 present examples of the vignette starting screen and main evaluation question with DSS support. The study was conducted completely anonymously; participants were only asked about their practice location (rural vs urban) and their gender.

### Statistical Analysis and Outcomes

A descriptive analysis was performed on both participant and case evaluation levels to identify potential trends. The binary outcome variables of interest for GDPs were “informing about the correlation between T2DM and periodontitis,” “recommending a GP visit to patients with high T2DM risk,” and “asking for the HbA_1c_ value of known T2DM patients.” We consider all these steps to be recommended actions, as all the cases either represent known T2DM or exhibit a high risk for T2DM. For GPs, the binary outcomes consisted of “informing about the correlation between T2DM and periodontitis” and “recommending a GDP visit to patients with high periodontitis risk.” We performed logistic regression models for all outcome variables on the case evaluation level. DSS assistance was used as binary independent variable. Subsequently, the model was run clustering the standard errors on the vignette level to account for intracluster correlation. *P* values lower than .05 were considered to indicate statistical significance. Statistical analyses were carried out using Stata/SE (version 14.2; StataCorp).

### Ethical Considerations

Study participants gave informed consent to participate in the survey, for anonymous data collection, and for the secondary analysis without additional consent. The study data are anonymous. Each participant who completed the survey received a financial compensation of EUR 20 (US $22.44). According to German law (Ärztliche Berufsordnung, §15[1]), ethical approval for this study was not requested.

## Results

### Descriptive Statistics

In total, 202 GDPs and 206 GPs participated in the study.

### GDPs Recommending a GP Visit

In all cases combined, the GDPs recommended a GP visit to patients in 41% (39/94) of occasions under DSS assistance, compared to 13% (69/512) of occasions without DSS assistance. Looking at the individual cases (Table 1) we see that this difference is much larger for the patients with high diabetes risk compared to those with diagnosed T2DM.

**Table 1:** General dental practitioners recommending a general practitioner visit.

Cases	Recommendations without DSS^a^ support, n/N (%)	Recommendations with DSS support, n/N (%)	*P* values^b^
Overall	69/512 (13)	39/94 (41)	<.001
A	15/90 (17)	12/18 (67)	<.001
B	12/89 (13)	9/13 (69)	<.001
C	13/81 (16)	9/19 (47)	.003
D	15/79 (19)	2/14 (14)	.68
E	9/87 (10)	4/13 (31)	.04
F	5/86 (6)	3/17 (18)	.10

^a^DSS: decision support system.

^b^Pearson chi-squared test.

Overall, the percentage of GDPs recommending a GP visit was highest for cases A and B, representing patients with clear periodontitis signs and exhibiting all common risk factors for T2DM.

### GPs Recommending a GDP Visit

Descriptive analysis showed that GPs were more likely to recommend a GDP visit under DSS assistance (20%, corresponding to 41 of 202 occasions) than without DSS assistance (3%, corresponding to 12 of 404 occasions). Looking at the individual cases, we can see that the difference is the highest for case C, representing the patient with the highest HbA_1c_ value (Table 2).

**Table 2:** General practitioners recommending a general dental practitioner visit.

Cases	Recommendations without DSS^a^ support, n/N (%)	Recommendations with DSS support, n/N (%)	*P* values^b^
Overall	12/404 (3)	41/202 (20)	<.001
A	4/133 (3)	8/69 (12)	.01
B	2/133 (2)	15/69 (22)	<.001
C	6/138 (4)	18/64 (28)	<.001

^a^DSS: decision support system.

^b^Pearson chi-squared test.

### GDPs Asking for HbA_1c_ Values

For GDP cases with known T2DM patients (cases C, D, E, and F), GDPs inquired about the HbA_1c_ value in 81% (51/63) of occasions under DSS assistance, compared to 51% (169/333) without DSS assistance. This difference is significant at the .05 level for all case vignettes except for case F (Table 3). This case represents the patient with T2DM and severe periodontitis but without the common behavioral risk factors, such as obesity, lack of physical activity, and poor diet.

**Table 3:** General dental practitioners asking for patients’ glycated hemoglobin value.

Cases	Recommendations without DSS^a^ support, n/N (%)	Recommendations with DSS support, n/N (%)	*P* values^b^
Overall	169/333 (51)	51/63 (81)	<.001
C	43/81 (53)	16/19 (84)	.01
D	35/79 (44)	11/14 (79)	.02
E	42/87 (48)	12/13 (92)	.003
F	49/86 (57)	12/17 (71)	.30

^a^DSS: decision support system.

^b^Pearson chi-squared test.

### Giving Advice on Common Risk Factors

With DSS assistance, GDPs advised their patients in 85% (80/94) of occasions on the correlation between T2DM and periodontitis and their common risk factors, compared to 77% (394/512) of occasions without DSS assistance. For GPs, a higher percentage (58%, corresponding to 236 of 404 occasions) advised the patients on common risk factors for periodontitis and T2DM without DSS support than with DSS support to 52% (105/202 occasions). However, the observed differences (with DSS vs without DSS) in giving advice on common risk factors were not statistically significant (*P*=.08 and *P*=.13, respectively).

### Logistic Regressions

The logistic regression models confirm the observed bivariate differences (Table 4). GDPs had 4.33 (95% CI 2.08-9.04) times higher odds of recommending a GP visit and 1.61 (95% CI 1.03-2.33) times higher odds of giving advice on the disease relationship when supported by the DSS. They had 3.22 (95% CI 1.63-6.35) times higher odds of asking for a patient’s HbA_1c_ value. GPs had 8.32 (95% CI 4.32-16.03) times higher odds of recommending a GDP visit under DSS assistance. There was no significant impact of DSS assistance on GP advice about common risk factors for T2DM and periodontal disease (odds ratio 0.77, 95% CI 0.53-1.98).

**Table 4:** Logistic regression results.

Outcomes	Odds ratio with decision support system (95% CI)	*P* value
GDP^a^ recommending a GP^b^ visit	4.33 (2.08-9.04)	≤.001
GDP advising on disease correlation	1.61 (1.03-2.33)	.04
GDP asking for glycated hemoglobin value	3.22 (1.63-6.35)	.001
GP recommending a GDP visit	8.32 (4.32-16.03)	≤.001
GP advising on common risk factors	0.77 (0.53-1.98)	.56

^a^GDP: general dental practitioner.

^b^GP: general practitioner.

## Discussion

### Principal Results

The findings of this study provide a robust proof of concept for the role of a digital DSS in enhancing integrated T2DM and periodontal care. The prototype DSS was found to result in GPs and GDPs having a 4 to 8 times higher chance of referring to each other. GDPs were also found to be more likely to advise patients with periodontitis or at high risk for T2DM on the correlation between the two conditions; they were also more likely to ask for HbA_1c_ values of known T2DM patients. These findings highlight the vast potential of a digital DSS for improving patient health outcomes and eventually reducing treatment costs at the medical-dental interface.

Digital DSSs based on evidence and rules represent a promising tool to improve the quality of care and patient outcomes [24]. DSSs based on electronic health records have also been successfully used to improve patients’ HbA_1c_ values [25]. Clinical DSSs have led to improved health outcomes when used in ambulatory and primary care to facilitate disease management [26]. We were able to confirm these findings using a case vignette methodology and show that a DSS could also be promising to drive the integration of primary and oral health care.

Along the lines of personally tailored medicine, our findings reveal valuable insights in the potential role of DSSs in tailoring the integrated management of T2DM and periodontitis according to specific patient characteristics. Under DSS assistance, different patient characteristics (represented by vignettes) were found to translate to distinct behavior changes among GDPs and GPs, thereby revealing some intriguing patterns. Among the case vignettes for GDPs, the largest effect of the DSS on GP recommendations was found for the cases with high T2DM risk, but without a T2DM diagnosis. This observation seems independent of the periodontal health of the patient. One potential explanation could be that GDPs assume that patients with known T2DM are already monitored by GPs with regular visits, and therefore do not need a recommendation for a GP visit. Since it is estimated that undiagnosed diabetes is a substantial problem [7,27], the DSS could play a role in earlier identification of undiagnosed T2DM patients and thereby aid earlier initiation of T2DM treatment if necessary.

While the overall recommendation rate of GPs to GDPs was very low, it significantly increased under DSS assistance. However, the average rate was still only 20%, which means that 4 of 5 rated case vignettes that were at risk of periodontitis were not recommended to go to the GDP. The participants in this study were unable to provide us with explanations for their choices, so we can only speculate why this happened. Potentially, GPs are less aware of the importance of the bidirectional relationship between T2DM and periodontitis. Potentially, they are more focused on the more established complications of T2DM, such as neuropathy, retinopathy, nephropathy, and cardiovascular disease. In addition, behavior change in clinical workflows is likely to evolve continuously over time. Considering that our case vignette study simulated only a short intervention period, the longer-term effects of DSS implementation may be larger than those observed in this study.

We also cannot fully explain why the DSS had relatively little impact on GPs in terms of counseling on common risk factors for T2DM and periodontitis. In addition to a low awareness of the relevance of periodontitis (also reflected in relatively low referral rates to GDPs—see above), one explanation could be that GPs consider a referral to a GDP as a surrogate (and easier) route of action in comparison to engaging in time-consuming counseling about common risk factors. Another explanation could be that the user interface design of the DSS was of limited appeal for GP participants. However, we are unable to verify these or any other potential explanations on the basis of our data. Further development of the DSS should pay close attention to the underlying reasons for the observed patterns of end-user behavior.

### Limitations

Our study has limitations. First, the nonrandom sampling of the participants prohibits generalization of our results. While this does not invalidate the trends detected in our results (ie, there is internal validity), the results of the vignette evaluations without the DSS cannot be considered as representative of the current status quo in Germany, thus limiting the external validity of our study. Similarly, our results cannot be generalized to other countries and settings. Notwithstanding this, the findings of this study provide a proof of concept for a digital DSS to integrate T2DM and periodontal care. Second, the survey had a closed-ended nature with limited answer options. Even though we tried to make all likely answer options available, the participants might have had valid reasons for not choosing our desired outcomes. The reasons for choosing the desired outcomes were unclear. Third, the size of our effects is likely influenced by the graphical representation of the DSS hints, which was limited by the options available in LimeSurvey. Potentially, a DSS interface designed for end-user applicability and smoothly customized for alignment with concrete workflows in practice software systems may lead to larger effect sizes. Fourth, in the absence of concrete clinical practice guidelines for the intersectoral treatment of T2DM and periodontitis, the specific actionable rules in the DSS were based on expert opinion and consensus. Upon availability of more concretely actionable clinical practice guidelines, the DSS’s rules could be adapted to closely match such guidelines.

### Comparison With Prior Work

The correlation between T2DM and periodontitis is reasonably well established [3-6,28]. Nevertheless, treatment of both conditions is still largely provided separately and without consideration for the other health condition. There is evidence for health benefits for patients with T2DM receiving periodontal treatment, such as better glycemic control [9,29,30] and lower rates of coronary heart disease [10]. In turn, these effects lead to financial benefits for health systems and affected individuals [31]. Finding ways to improve intersectoral cooperation and making sure patients receive appropriate care for general and dental conditions deserves careful attention. This DSS could be helpful in earlier identification and integrated management of patients at risk of periodontitis and T2DM and provides a blueprint for other oral-systemic interactions, such as periodontitis and cardiovascular disease.

### Conclusions

To summarize, our findings highlight that a digital DSS offers a viable path to improve patient-centered care of individuals with T2DM and periodontitis. The evaluated DSS prototype represents a uniquely relevant starting point for DSS innovation at the medical-dental interface. Future updating and testing is warranted to continuously enhance the functionalities of the DSS in terms of interoperability with various types of data sources and diagnostic devices; incorporation of other (oral) health dimensions; application in various settings, including telemedicine; further customization of end-user interfaces; and smooth alignment with dynamically evolving clinical workflows. In addition, it will be highly relevant to keep monitoring developments in the intersectoral cooperation between GPs and GDPs in order to customize the DSS for value and applicability and avoid burdening clinicians with wasteful DSS information about routes of action that they may already be performing—potentially even better—without DSS assistance.

## Abbreviations

DSS: decision support system
GDP: general dental practitioner
GP: general practitioner
HbA_1c_: glycated hemoglobin
T2DM: type 2 diabetes mellitus

## Data Availability

The data sets generated during and/or analyzed during this study are available from the corresponding author on reasonable request.
